# Novel Use of Clonidine Patch to Treat Tizanidine Withdrawal

**DOI:** 10.7759/cureus.54831

**Published:** 2024-02-24

**Authors:** Aaron B Deutsch, Clare F Hartman, Curtis P Flaherty, Natalie E Ebeling-Koning, Gillian A Beauchamp, Kenneth D Katz

**Affiliations:** 1 Department of Emergency and Hospital Medicine, Lehigh Valley Health Network/University of South Florida (USF) Morsani College of Medicine, Allentown, USA

**Keywords:** clonidine taper, transdermal clonidine, tizanidine withdrawal, alpha-2 agonist, tizanidine use disorder

## Abstract

Tizanidine is commonly prescribed for muscle spasticity and pain. Yet, withdrawal is rarely reported. Tizanidine stimulates presynaptic α-2 adrenergic and imidazoline receptors decreasing norepinephrine release. Abrupt cessation can cause withdrawal. Current treatment strategies include tapering oral tizanidine or substituting oral clonidine. A 52-year-old male with a history of hypertension, diabetes, coronary artery disease, and chronic back pain presented with altered mental status, agitation, hypertensive emergency (blood pressure: 250/145 mmHg), and tachycardia. The patient had been prescribed tizanidine for chronic back pain for two years and had recently run out with suspicion of misuse. Tizanidine withdrawal was diagnosed, and he improved with 0.1 mg oral clonidine three times daily weaned over five days while hospitalized. One month later the patient was admitted for persistent hypertension, tachycardia, diaphoresis, and anxiety. Alpha-2 agonist withdrawal was again diagnosed. Utilizing a clonidine patch taper may offer a reasonable approach in patients with tizanidine withdrawal.

## Introduction

Tizanidine is a presynaptic α-2 adrenergic and imidazoline receptor agonist that decreases norepinephrine release [1]. Commonly prescribed for pain and muscle spasticity, tizanidine may also treat opioid withdrawal [2]. Abrupt cessation of chronic usage may cause clinically significant, yet likely underreported, α-2 agonist withdrawal manifesting as tachycardia, hypertension, tremor, spasms, and anxiety [3]. Current treatment strategies for both management of opioid withdrawal and misuse of α-2 agonists include tapering the α-2 agonist or substituting an equivalent agent (e.g., oral clonidine or tizanidine) due to the similar mechanisms of action [4-8].

Clonidine is an α-2 adrenergic agonist that decreases sympathetic output [9]. It is prescribed as an antihypertensive, as well as for treatment of both opioid withdrawal and chronic pain [9-11]. Formulations include both oral tablets and transdermal patches. Therapeutic transdermal clonidine concentrations are reached two to three days following initiation, after which a therapeutic concentration is maintained, eliminating the peaks and troughs that occur with oral administration. Even after patch removal, residual subcutaneous clonidine continues to act as an auto-taper due to a depot-like effect, leading to reduced rebound hypertension and fewer side effects compared to the oral formulation [12].

Utilizing alternatives to opioid medications for pain management, such as α-2 agonists, is critical in the ongoing opioid overdose crisis. However, these alternatives may themselves produce adverse effects, toxicity, and withdrawal if not properly managed. Patients with substance use disorders may be at particularly high risk. Therefore, recognizing both drug-induced toxidromes and withdrawal states remains critical. A novel approach to the management of tizanidine withdrawal using a transdermal clonidine taper is presented.

This study in part was previously presented as an abstract at the 2023 Pennsylvania College of Emergency Physicians Scientific Assembly (May 5, 2023, Pocono Manor, PA), and the 2023 North American Congress of Clinical Toxicology (September 29, 2023, Montreal Quebec, Canada).

## Case presentation

A 52-year-old male with a history of hypertension, diabetes, coronary artery disease, and chronic back pain presented with altered mental status, agitation, hypertensive emergency with presenting blood pressure (BP) of 250/145 mmHg and tachycardia, with initial heart rate (HR) of 136 beats per minute. Diagnostic testing revealed acute kidney injury and a non-ST elevation myocardial infarction (Table 1). The patient had been taking tizanidine for chronic back pain for the past two years. He was prescribed 6 mg up to three times daily as needed (maximum 18 mg); however, per discussion with the patient’s wife, he frequently called for early refills. Prior to his first hospital admission, the patient had been taking 30-34 mg daily. The patient did not have a history of illicit drug use. Toxicology drug screening was performed (Table 2). Of note, the patient had received 5 mg of diazepam in the emergency department prior to the collection of the toxicological drug screening samples. Tizanidine withdrawal was diagnosed, and his adrenergic signs and symptoms improved with 0.1 mg oral clonidine three times daily weaned over five days while hospitalized. The patient’s tizanidine prescription was discontinued.

**Table 1:** Patient abnormal lab values on initial presentation. CBC: complete blood count; WBC: white blood cells; RBC: red blood cells; BUN: blood urea nitrogen; CK: creatine kinase; HS: high sensitivity

Test	Result	Normal range	Units
CBC			
WBC	22.6	4.0-10.5	thousand/mm³
RBC	5.51	4.00-5.40	million/mm³
Platelet count	404	140-350	thousand/mm³
Differential			
Absolute neutrophils	19.2	1.8-7.8	thousand/mm³
Absolute monocytes	1.3	0.3-1.0	thousand/mm³
Chemistry			
Glucose	331	70-110	mg/dL
BUN	29	7-18	mg/dL
Creatinine	1.34	0.60-1.30	mg/dL
Carbon dioxide	21	22-28	mmol/L
Anion gap	13	3-11	N/A
Protein (total)	9.1	6.4-8.3	g/dL
Total bilirubin	1.4	0.2-1.0	mg/dL
Alkaline phosphatase	125	32-91	U/L
CK (total)	229	52-200	U/L
Lactate	3.5	0.5-2.2	mmol/L
Cardiac markers			
HS troponin (hour 0)	59	<21	ng/L
HS troponin (hour 1)	60	<21	ng/L
Troponin I (HS)	353	<80	ng/L
Urinalysis			
Protein (urine)	>500	Negative	mg/dL
Glucose (urine)	50-149	Negative	mg/dL
Ketone (urine)	5-19	Negative	mg/dL
Blood (urine)	0.20-0.99	Negative	mg/dL
Leukocytes esterase	25	Negative	/µL
Bacteria	1+	Negative	N/A

**Table 2:** Toxicological drug screening results. IA: immunoassay; LC-MS: liquid chromatography/mass spectrometry; LC/QTOF/MS: liquid chromatography/quadrupole time of flight mass spectroscopy

Test	First admission	Second admission
Urine drug screen (IA)		
Amphetamines	Negative	Negative
Barbiturates	Negative	Negative
Benzodiazepine	Positive (unconfirmed)	Negative
Cannabinoids	Negative	Negative
Cocaine	Negative	Negative
Opiates	Negative	Negative
Phencyclidine	Negative	Negative
Oxycodone	Negative	Negative
Drug confirmation testing (LC-MS)		
Diazepam	Negative	N/A
Lorazepam	Negative	N/A
Nordiazepam	Negative	N/A
Oxazepam	Negative	N/A
Temazepam	Negative	N/A
Hydroxymidazolam	Negative	N/A
7-Aminoclonazepam	Negative	N/A
2-OH-ethylflurazepam	Negative	N/A
Alpha-OH-alprazolam	Negative	N/A
Venous drug screen (LC/QTOF/MS)		
Diazepam	Positive (unconfirmed)	N/A
Diphenhydramine	Positive (unconfirmed)	N/A
Nordiazepam	N/A	Positive (unconfirmed)

One month later the patient was re-admitted for hypertension (BP: 161/129 mmHg), tachycardia (HR: 120), diaphoresis, and anxiety. Toxicology drug screening was performed (Table 2). Due to the discontinuation of his tizanidine prescription after the previous hospitalization, he obtained tizanidine online without a prescription and continued taking 30 mg daily. He had again run out of medication prior to the hospital presentation. Alpha-2 agonist withdrawal was again diagnosed and complete abstinence from the medication was re-iterated. Substance use counseling was recommended, and a 0.3 mg clonidine patch was initiated, with the goal of improving compliance and providing gradual withdrawal treatment. Clonidine patches were prescribed in a tapering fashion for a total of three weeks (0.2 mg/day week two, 0.1 mg/day week three). After inpatient patch initiation, the adrenergic signs and symptoms improved, and the patient had normal vitals at a follow-up appointment three days after discharge.

## Discussion

Utilizing tapering doses of transdermal clonidine may offer a reasonable approach for the treatment of patients experiencing α-2 agonist withdrawal from medications such as tizanidine. Use of the transdermal patch compared to oral formulations has several potential benefits. First, it may improve compliance by avoiding the necessity of multiple daily doses. Second, the pharmacokinetics of a transdermal formulation are favorable because peaks and troughs of medication concentration are eliminated while providing an auto-tapering of dosing via a depot-like effect. Evidence of continued effect and potential prevention of recurrence of withdrawal symptoms is supported by literature suggesting that patients using the patch had less rebound hypertension compared to the oral formulation [12].

Utilizing opioid alternatives for pain management is critical in the ongoing opioid crisis. Studies have shown α-2 agonists may help both with management of opioid withdrawal and as a non-opioid modality in managing chronic pain [11,13]. Compared to methadone, opioid withdrawal symptoms occurred and resolved faster in patients prescribed α-2 agonists [13]. These therapies could be useful in transitioning patients from opioids to opioid alternatives while providing both relief for their underlying painful conditions and management of withdrawal.

However, as demonstrated in this study, α-2 agonists are not exempt from the risk of dependence and withdrawal, and patients with a history of substance use disorders may be at risk for misuse of this class of medications [14]. While the risk of dependence may be minimal at low tizanidine doses (3-6 mg) [15], consuming α-2 agonists, such as tizanidine, at high doses can manifest side effects that may be considered desirable, such as sedation [16]. As opioid prescribing practices change, and opioid alternatives are utilized more frequently for patients with chronic pain conditions, it is possible that patients experiencing withdrawal from adjunctive non-opioid medications such as α-2 agonists may present more frequently for medical care [17]. Alpha-2 agonist withdrawal should remain in the differential of patients exhibiting otherwise unexplained adrenergic signs and symptoms.

## Conclusions

Transdermal clonidine was administered to both uniquely and successfully treat a patient suffering from tizanidine withdrawal. Improving patient compliance and ease of administration with transdermal formulations may be beneficial in the treatment of α-2 agonist withdrawal.
